# Consequences of In Utero Zika Virus Exposure and Adverse Pregnancy and Early Childhood Outcomes: A Prospective Cohort Study

**DOI:** 10.3390/v14122755

**Published:** 2022-12-10

**Authors:** Rebecca Grant, Olivier Flechelles, Narcisse Elenga, Benoît Tressières, Stanie Gaete, Jean-Christophe Hebert, Bruno Schaub, Felix Djossou, Adeline Mallard, Lucetta Delver, Catherine Ryan, Anna L. Funk, André Cabié, Arnaud Fontanet, Bruno Hoen

**Affiliations:** 1 Emerging Disease Epidemiology Unit, Institut Pasteur, 75015 Paris, France; 2 Sorbonne Université, 75006 Paris, France; 3 Centre Hospitalier Universitaire de la Martinique, 97261 Fort-de-France, France; 4 Centre Hospitalier de Cayenne, Cayenne 97306, French Guiana; 5 Centre d’Investigation Clinique Antilles—Guyane, 97142 Pointe-à-Pitre, France; 6 Karubiotec™, Centre Hospitalier Universitaire de la Guadeloupe, 97142 Pointe-à-Pitre, France; 7 Centre Hospitalier de Basse-Terre, 97109 Basse-Terre, France; 8 Centre Hospitalier Universitaire de la Guadeloupe, 97142 Pointe-à-Pitre, France; 9 Pathogenesis and Control of Chronic and Emerging Infections, Université de Montpellier, Université des Antilles, INSERM, EFS, 34394 Montpellier, France; 10 Centre d′Investigation Clinique 1424, INSERM, 97261 Fort-de-France, France; 11 Conservatoire National des Arts et Métiers, 75003 Paris, France

**Keywords:** Zika, congenital Zika syndrome, pregnancy outcomes, congenital abnormalities, child development

## Abstract

We aimed to describe adverse pregnancy outcomes among women who had symptomatic, RT-PCR-confirmed ZIKV infection and early childhood outcomes among their infants. We enrolled pregnant women with symptomatic, RT-PCR-confirmed ZIKV infection in a prospective cohort study, and their infants in a prospective pediatric cohort study. We defined adverse pregnancy and early childhood outcomes based on selected neurologic, ophthalmologic, auditory, musculoskeletal, and anthropometric abnormalities. We used RT-PCR and serologic tests to determine the ZIKV infection status of the child. Between 10 March and 24 November 2016, we enrolled 546 pregnant women with RT-PCR-confirmed ZIKV infection. The overall risk of adverse pregnancy and early childhood outcomes possibly related to in utero ZIKV exposure was 15.7% (95% CI: 12.8–19.0), distributed as follows: 3.6% (95% CI: 2.3–5.6) severe sequelae or fatality; 2.7% (95% CI: 1.6–4.5) major abnormalities; 9.4% (95% CI:7.1–12.2) mild abnormalities. The risk of severe sequelae or fatality was higher when ZIKV infection occurred during the first trimester (7.0%), compared to the second (2.7%) or third trimester (1.4%) (*p* = 0.02). Among the infants for whom ZIKV infection status could be determined, the vertical transmission rate was 3.0% (5/167) (95% CI: 1.1–7.2). Among pregnant women with symptomatic, RT-PCR-confirmed ZIKV infection, severe or major pregnancy or early childhood outcomes were present in 6.3% of fetuses and infants. Severe outcomes occurred more frequently in fetuses and infants whose mothers had been infected in the first trimester.

## 1. Introduction

In utero ZIKV infection is known to cause congenital neurologic abnormalities, vision and hearing defects, motor dysfunction, and developmental delays [1,2,3,4,5,6,7]. Descriptions of abnormalities caused by in utero ZIKV infection have largely focused on pregnancy and neonatal outcomes. Abnormalities in early childhood have also been described, including developmental delays and anthropometric abnormalities, including microcephaly, occurring in the post-natal period [6,7,8,9] and live born children with congenital Zika syndrome have been shown to be at increased risk of mortality, as compared to those without congenital Zika syndrome [10]. However, the evidence on the risk of adverse outcomes in early childhood in infants born to women infected with ZIKV during pregnancy—and not just those with congenital Zika syndrome—is limited [11].

The ZIKV epidemic in French Territories in the Americas (French Guiana, Guadeloupe, and Martinique) began in early 2016. In a cohort study of 546 pregnant women with symptomatic, RT-PCR-confirmed ZIKV infection, the risk of adverse pregnancy outcomes was estimated to be 7.0%, with a greater risk when the ZIKV infection occurred during the first trimester (12.7%), compared to the second trimester (3.6%) or the third trimester (5.3%) [12]. A subsequent pediatric cohort study was established to follow the infants born to these women up to 24 months of age. The objective of this study was to describe the outcomes up to 24 months of age of the infants born to women who had symptomatic RT-PCR-confirmed ZIKV infection during pregnancy.

## 2. Materials and Methods

To study the impact of in utero ZIKV exposure on pregnancy and early childhood outcomes, the ZIKA-DFA-FE (pregnant women) and ZIKA-DFA-BB (newborns) prospective cohort studies were implemented in Guadeloupe, Martinique, and French Guiana in 2016 during the ZIKV epidemic. A key component of the ZIKA-DFA-FE and ZIKA-DFA-BB cohort studies was the prospective and standardized clinical follow-up, which lasted until the end of pregnancy in the case of the pregnant women cohort study, and until 24 months of age, in the case of the newborn cohort study.

### 2.1. Criteria for Inclusion in Analysis

In accordance with the guidelines of the French High Council for Public Health and the French National College of Gynaecologists and Obstetricians that were issued on 28 July 2015 and 5 February 2016, respectively, whenever a pregnant woman presented to an outpatient clinic or emergency department of a participating center with symptoms consistent with acute ZIKV infection, she underwent a clinical examination, and blood and urine specimens were obtained to confirm a recent ZIKV infection.

Women were included in this analysis if they met all of the following criteria at the time of their enrolment in the ZIKA-DFA-FE cohort study: (1) ongoing pregnancy; (2) clinical symptoms consistent with acute ZIKV infection, with at least one symptom of pruritic rash, fever, conjunctival hyperemia, arthralgia, or myalgia; (3) laboratory confirmation by RT-PCR of recent ZIKV infection performed on a specimen of blood, urine, or both [12]. All live-born infants born to women meeting the eligibility criteria described above were included in the analysis of adverse early childhood outcomes.

### 2.2. Cohort Follow-Up

All neonates/fetuses underwent routine clinical examination at the end of pregnancy. Live-born infants were eligible for inclusion in the pediatric cohort study if they were born at or after 35 weeks gestational age. Eligible infants included in the prospective pediatric cohort underwent further standardized clinical examinations at 2, 4, 9, 18, and 24 months of age. Additional examinations were offered between 4–9 months, 9–18 months, and 18–24 months of age, if the infant had missed any previous clinical examination. Additional imaging (cerebral ultrasonography) evaluations and clinical (ophthalmologic and auditory) examinations were conducted primarily at birth, or at any point in the follow-up period if the additional imaging or examinations were not performed at birth. Further magnetic resonance imaging (MRI) was performed at any point in the follow-up period, upon indication of the attending pediatric clinician.

For more complete follow-up of those who declined participation in the pediatric cohort, or those who were lost to follow-up across the 24-month follow-up period, medical registries in Guadeloupe, Martinique, and French Guiana were consulted to determine the infant’s vital status at 24 months of age. For any death, the date and cause of death were recorded. Any abnormal developmental outcomes at 24 months of age that were noted in the medical records were also recorded.

### 2.3. Adverse Early Childhood Outcomes

As in utero ZIKV infection has been associated with neurological, visual, auditory, and anthropometric abnormalities [1,3,5], as well as developmental delays [2,3,4,5,6,7], we considered the following abnormalities and adverse developmental outcomes at any time in the 24-month follow-up period, as defined by Rice et al. [8]:

- Abnormal auditory examination which included otoacoustic emissions or automated auditory evoked potential assessments;
- Abnormal ophthalmologic examination, which included RetCam wide-field retinal imaging (Shuttle, Clarity Medical Systems, Pleasanton, USA) for fundus imaging and abnormalities in vitreous, choroid, retina and optic disc; or visual impairment documented by visual screening test;
- Clinician-documented seizures, excluding febrile seizures;
- Body tone abnormalities, hypertonia or hypotonia, suspicion or diagnosis of cerebral palsy;
- Musculoskeletal or motor abnormalities, dyskinesia or dystonia, documented by the clinician;
- Swallowing abnormalities, documented by instrumented or non-instrumented evaluation;
- Post-natal onset microcephaly, defined as the two most recent head circumference measurements < 3rd percentile for age and sex based on the World Health Organization’s Child Growth Standards, or by a downwards trajectory of head circumference percentiles, with the most recent measurement < 3rd percentile;
- Possible neurodevelopmental delays, through administration of three pediatric neurodevelopment evaluation tools administered to a parent/legal guardian at the time of the 24-month pediatric consultation [13]: a 30-item parent-reported screening test, Ages and Stages Questionnaire-III (ASQ) [14], previously validated in France [15], to identify toddlers at risk for developmental delay; a 23-item parent-reported screening test, Modified Checklist for Autism on Toddlers (M-CHAT), to identify toddlers at risk for behavior disorder [16]; the French MacArthur-Bates Communicative Development Inventories (Inventaires français du développement communicative—IFDC) to assess French language acquisition [17].

In addition, we also considered abnormal transfontanelle cerebral ultrasound, and/or MRI at any time in the 24-month follow-up period.

### 2.4. ZIKV Infection Status

As all women had RT-PCR-confirmed ZIKV infection during pregnancy, all infants were considered exposed to ZIKV in utero. A cord blood sample was collected at delivery, or a serum sample from the newborn within the first 10 days of life. In addition, a serum sample was collected during one of the routine clinical examinations after 9 months of age. These samples were used to determine the ZIKV infection status of the infant. An infant was considered to have had in utero ZIKV infection if ZIKV RT-PCR was positive at birth in the infant’s blood or urine, in placenta or in amniotic fluid; or if anti-ZIKV IgM antibodies were detected (EuroImmun ELISA or in-house MAC ELISA in French Guiana [18]) in the cord blood or serum collected within the first 10 days of life; and/or if anti-ZIKV IgG antibodies were detected (EuroImmun ELISA or in-house MAC ELISA in French Guiana [18]) in the serum sample beyond 9 months of age and the date of birth was posterior to the end of the ZIKV epidemic, as determined by the French Public Health Agency: 11 September 2016 in French Guiana, 25 September 2016 in Guadeloupe, and 16 October 2016 in Martinique. An infant was considered as having no evidence of in utero ZIKV infection if the above infant tests were negative. If samples or results were missing or equivocal, the infant’s ZIKV infection status was considered undetermined.

### 2.5. Statistical Analysis

The characteristics of women and infants included and not included in the pediatric cohort study were compared using Student’s t-test for continuous variables and Pearson’s chi-squared test for categorical variables. Adverse pregnancy and early childhood outcomes were categorized based on severity and, in the case of early childhood outcomes, persistence, in consultation with the study’s referent pediatrician, and compared by trimester of maternal ZIKV infection using Pearson’s chi-squared test. For those included in the pediatric cohort study, adverse early childhood outcomes were also compared by the child’s ZIKV infection status using Fisher’s exact test for categorical variables due to the small number of participants in the group with evidence of ZIKV infection. For all statistical analyses, *p* < 0.05 was considered statistically significant. All statistical analyses were performed using R, version 3.6.1.

## 3. Results

**Figure 1:**
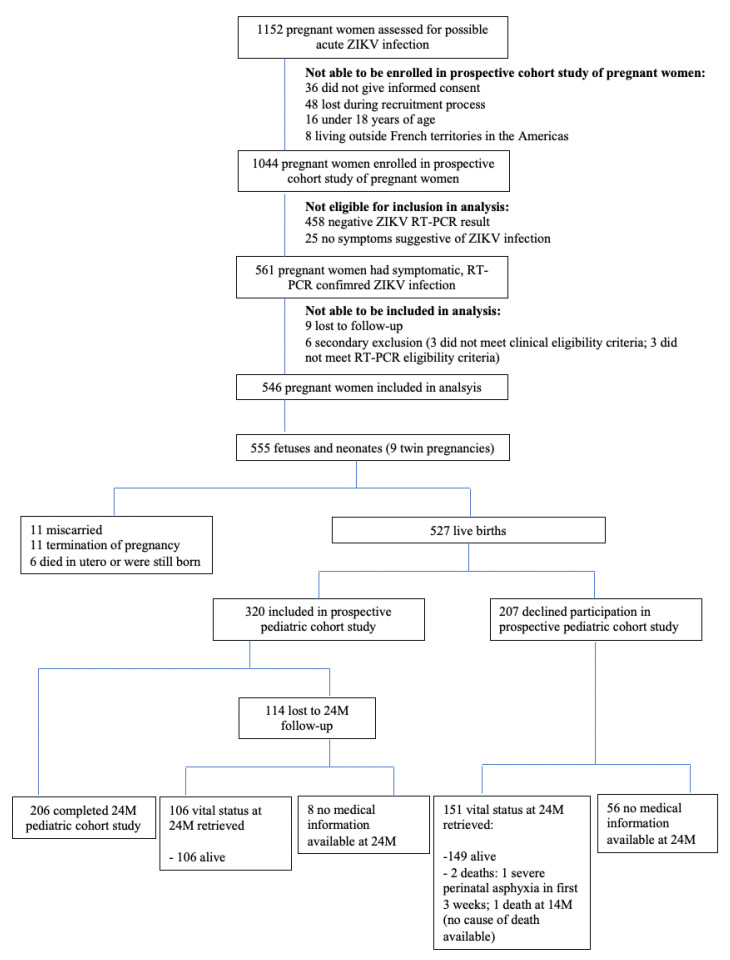
Inclusion of women with symptomatic RT-PCR-confirmed ZIKV infection during pregnancy in ZIKA-DFA-FE cohort study and inclusion of liveborn infants in ZIKA-DFA-BB cohort study.

Between 2 March 2016 and 24 November 2016, 1152 pregnant women were assessed for possible acute ZIKV infection. Of these, 546 women with symptomatic, RT-PCR-confirmed ZIKV infection during pregnancy were included in this analysis. From the 546 pregnant women, there were 555 fetuses or newborns (nine twin pregnancies). Of these, there were 28 (5.0%) pregnancy losses: spontaneous or voluntary abortions, medical terminations of pregnancy, or stillbirths. Among the 527 live births, 320 were included in the prospective cohort study, of which 206 completed 24 months of follow-up. Additional information from medical registries was obtained at 24 months of age for 151 infants whose parent/guardians declined their inclusion in the prospective cohort study at birth and from 106 infants who were included in the pediatric cohort study but who were lost to follow-up during the 24-month study period (Figure 1).

Table 1 describes the characteristics of the 546 women and 555 fetuses/newborns included in this analysis. Supplementary Table S1 compares the maternal and neonatal characteristics of the women whose infant was included in the pediatric cohort study and of the women whose infant was not included in the pediatric cohort study. The only difference between the two groups was the place of residence, with a greater proportion of infants included in Martinique and Guadeloupe, compared to French Guiana (*p* < 0.001).

### 3.1. Adverse Pregnancy Outcomes

The risk of adverse pregnancy outcomes in this cohort has been previously published [12]. Briefly, the overall risk of neurologic and ocular defects possibly associated with maternal ZIKV infection that were evident at birth was 7.0% (95% CI 5.0–9.5) and the risk of congenital Zika syndrome was 3.1% [12], using a definition provided by Moore C et al. [1]: severe microcephaly (head circumference more than 3 SD below the mean), brain abnormalities with a specific pattern of damage (e.g., calcifications, ventriculomegaly, or cortical malformations), damage to the back of the eye, joints with limited range of motion (e.g., clubfoot), or hypertonia that restricts body movement (e.g., arthrogryposis). Neurologic and ocular abnormalities were more common when maternal ZIKV infection had occurred during the first trimester (12.7%), compared to the second (3.6%) or third trimester (5.3%) (*p* = 0.001) [12].

### 3.2. ZIKV Infection Status

Of the 252 infants tested, 3 had a positive RT-PCR ZIKV result at birth in a blood, urine, placenta, or amniotic fluid sample. A total of 169 cord blood or newborn serum samples collected during the first 10 days of life were available for anti-ZIKV serologic testing: 60 from infants born to women infected in the first trimester, 84 from infants born to women infected in the second trimester, and 25 from infants born to from women infected in the third trimester. None of these available samples were positive for anti-ZIKV IgM antibodies. Anti-ZIKV IgG antibodies were detected in 11 of 182 (6.0%) serum samples collected from infants after 9 months of age. According to the diagnostic criteria described in the Methods, we were able to classify 5 (1.6%) children as having evidence of ZIKV infection, 162 (51.9%) as having no evidence of ZIKV infection, and the remaining 153 (46.6%) as having unknown/undetermined ZIKV infection status. Among the infants for whom ZIKV infection status could be determined, we found a vertical transmission rate of 3.0% (95% CI: 1.1–7.2).

### 3.3. Adverse Early Childhood Outcomes

Of the 320 infants who were included in the pediatric cohort study, 272 (85.0%) completed additional auditory examinations; 261 (81.6%) completed additional ophthalmologic examinations; 216 completed transfontanelle cerebral ultrasonography evaluations (67.5%); and 76 (23.8%) were referred for and completed an MRI examination. Table 2 shows the selected adverse early childhood outcomes at 2, 4, 9, 18, and 24 months of age by ZIKV infection status among those included in the pediatric cohort study. We did not consider neurodevelopmental delays, as not all children completed the neurodevelopment assessment at 24 months of age. Overall, we found that across the 24-month follow-up period, 60% of those with in utero ZIKV infection, 26.5% of those with no in utero ZIKV infection, and 23.1% of those with unknown/undetermined status had at least one of the selected abnormalities possibly linked to in utero ZIKV exposure, with most abnormalities reported at the first visit at 2 months of age and not again at subsequent follow-up consultations (Table 2).

Of the 151 infants not included in the pediatric cohort due to parents/guardians declining their participation, consultation of medical registries indicated that there were two deaths in early childhood: one due to severe perinatal asphyxia and occurring three weeks after birth; the other at 14 months of age, but the cause of death was not reported. With respect to selected abnormalities related to in utero ZIKV exposure, in non-standardized clinical assessments, three infants had vision impairment, and one had impaired hearing. Of the 106 infants who did not complete 24 months of follow-up as part of the pediatric cohort study, consultation of medical registries indicated that there had been no deaths during the follow-up period. With respect to selected abnormalities related to in utero ZIKV exposure, in non-standardized clinical assessments, vision impairment had been reported for three infants, and one infant had experienced seizures.

When considering adverse pregnancy and early childhood outcomes likely related to in utero ZIKV exposure (Table 3B), we found the overall risk to be 15.7% (95% CI: 12.8–19.0): 3.6% (95% CI: 2.3–5.6) severe sequelae or fatality; 2.7% (95% CI: 1.6–4.5) major abnormalities; 9.4% (95% CI: 7.1–12.2) mild abnormalities. Among the adverse pregnancy and early childhood outcomes likely related to in utero ZIKV exposure, we found the risk of severe sequelae or fatality to be higher when ZIKV infection had occurred during the first trimester (7.0%), compared to the second (2.7%) or third trimester (1.4%) (*p* = 0.02).

**Figure 2:**
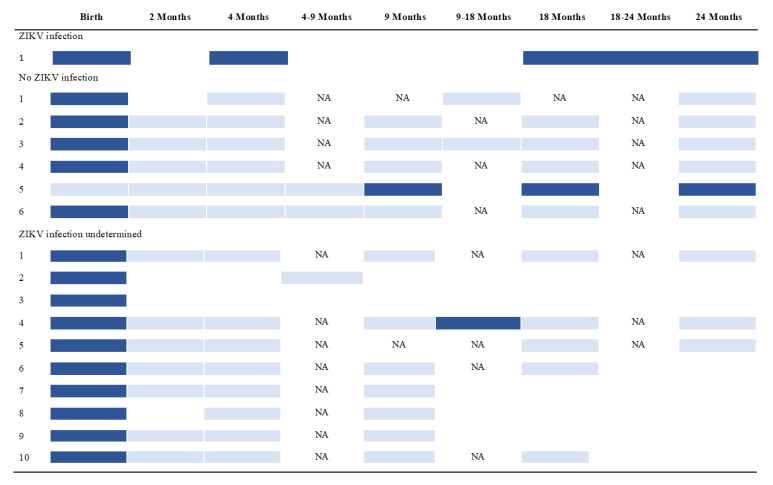
Evolution of microcephaly among 17 children included in the ZIKA-DFA-BB pediatric cohort study with microcephaly detected at birth or in the post-natal period. Legend: dark blue = microcephaly; light blue = absence of microcephaly; NA = pediatric consultation not conducted, no information on head circumference available—in the absence of head circumference measurement, microcephaly at the prior visit was assumed to persist until head circumference was measured at a subsequent visit.

Figure 2 shows the evolution of microcephaly among 17 children with microcephaly detected either at birth or in the post-natal period. We adopted a conservative approach, by which, in the absence of measurement (as indicated by the NA), microcephaly was assumed to persist until head circumference was measured at a subsequent study visit. Of the seventeen children, one with evidence of ZIKV infection had persistent microcephaly across the duration of follow-up. Importantly, 14 of the 16 children with anthropometric data available after birth showed resolution of microcephaly across the 24-month period of follow-up. Of these, one infant had recurrent and transient microcephaly between 9 and 18 months of age, but which was no longer detectable at 24 months of age.

## 4. Discussion

We have been able to describe the risk of adverse pregnancy and early childhood outcomes during the ZIKV epidemic in Guadeloupe, Martinique, and French Guiana in 2016. Among 546 women with symptomatic, RT-PCR-confirmed ZIKV infection during pregnancy, we found an overall risk of fetal or infant neurological and ocular defects possibly associated with maternal ZIKV infection to be 7% (95% CI 5.0–9.5), and the risk of congenital Zika syndrome to be 3.1% [12]. Among the 320 infants included in the pediatric cohort study, 79 (24.7%, 95% CI: 20.1–29.9) had at least one of the selected abnormalities possibly related to in utero ZIKV exposure in the post-natal period, but the majority of these were transient and mild abnormalities. When considering adverse pregnancy and early childhood outcomes likely related to in utero ZIKV exposure among the 555 fetuses and infants, we found the overall risk to be 15.7% (95% CI: 12.8–19.0): 3.6% (95% CI: 2.3–5.6) severe sequelae or fatal outcome; 2.7% (95% CI: 1.6–4.5) major abnormalities; 9.4% (95% CI: 7.1–12.2) mild abnormalities.

This study quantifies the risk of adverse pregnancy and early childhood outcomes, including an assessment of severity, using a standardized clinical assessment in a cohort of women with RT-PCR-confirmed ZIKV infection during pregnancy, complemented with information from non-standardized clinical assessments for those not included in the follow-up pediatric cohort study. The prospective mother-child cohort study design among women with symptomatic, RT-PCR-confirmed ZIKV infection allowed us to follow the evolution of clinical conditions from time of maternal ZIKV infection to 24 months of age. The categorization of the severity and persistence of abnormalities allowed us to distinguish infants with single occurrence, mild abnormalities from those with severe sequalae and impaired early childhood development. We were also able to show severe sequelae or fatal outcomes to be more frequent when maternal ZIKV infection occurred in the first trimester (7.0%), compared to the second (2.7%) or third trimester (1.4%) (*p* = 0.02). This is a particularly important consideration for clinical decisions regarding pregnancy management in the context of ZIKV infection.

Our results regarding the risk of selected abnormalities possibly related to in utero ZIKV exposure in the post-natal period are largely similar to an individual participant data meta-analysis of cohort studies from Brazil of pregnant women with RT-PCR-confirmed ZIKV infection during pregnancy [19]. Among live born infants, the risk for microcephaly at birth or first evaluation was 2.6% and 4.0% at any time during follow-up into early childhood, the risk for neuroimaging abnormalities was 7.9%, the risk of functional neurological abnormalities was 18.7%, the risk of ophthalmic abnormalities was 4.0%, the risk of auditory abnormalities was 6.4%, the risk of arthrogryposis was 0.6%, and the risk of dysphagia was 1.5% [19]. Similarly, Rice M et al. [8] found that among 1450 children over the age of 1 year and born to women with ZIKV infection during pregnancy in the United States of America who had non-standardized follow-up care reported at any time after 14 days of life, 87 (6%) had at least 1 ZIKV-associated birth defect, and 203 (14%) had a ZIKV-associated birth defect, neurodevelopmental abnormality possibly associated with congenital ZIKV infection, or both.

Nonetheless, there remains heterogeneity across individual studies. Among 130 children with antenatal exposure to ZIKV in Brazil, 45 had at least one abnormal finding on brain imaging, eye, neurological, or neurodevelopmental assessments in the first year of life [20]. In a separate cohort study in Brazil, Lopes Moreira ME et al. [21] followed 182 children born to women with symptomatic RT-PCR-confirmed ZIKV infection and offered systematic neuroimaging, complete eye examinations, and assessments of brainstem auditory evoked responses to all children. Among those who underwent both neurodevelopmental and neuroimaging assessments, 39/115 (34%) had abnormal findings, of which 20 were nonspecific hypersignal findings on MRI.

Heterogeneity also persists across individual studies in the rate of vertical transmission. All infants included in our analysis were born to women with symptomatic, RT-PCR-confirmed ZIKV infection during pregnancy. As such, we assume that all infants were exposed to ZIKV in utero. For the ZIKV infection status of the infants, among those for whom in utero ZIKV infection status could be determined, we found a vertical transmission rate of 3.0% (5/167) (95% CI: 1.1–7.2). Our estimate is close to the 4% estimate in 1450 children born to women enrolled in a registry-based study with confirmed or possible ZIKV infection during pregnancy in territories of the United States [8], as well as to the 7% estimate among 295 neonates born to women with confirmed or probable ZIKV infection during pregnancy in New York City, using ELISA immunoassay [22]. However, the inclusion in these two studies of women with possible or probable ZIKV infection, rather than RT-PCR-confirmed ZIKV infection, during pregnancy, likely underestimates the rate of vertical transmission in these two studies. Among 130 children with antenatal exposure in Brazil, 84 (65%) had at least one positive result in sera or urine tested by RT-PCR and IgM assays [20]. However, this percentage dropped to 33% beyond 3 months of age, and concordance between RT-PCR and IgM was only 52%. Furthermore, the study design did not effectively exclude the possibility of postnatal infection, which likely overestimates the vertical transmission rate. The most comprehensive assessment of the rate of vertical transmission comes from systematic RT-PCR and serologic testing on multiple tissue samples, including amniotic fluid, fetal and neonatal blood, cerebrospinal fluid, urine, and placenta, from 291 fetuses and newborns in French Guiana [23]. This study found the rate of vertical transmission to be 26.1%, revised to 18% when placental samples were not considered because of the risk of contamination from maternal viremia, which is considerably higher than our own estimate. In a subsequent prospective follow-up of newborns to 3 years of age in French Guiana, confirmed congenital ZIKV infection at birth was associated with higher risks of adverse neonatal and early infantile outcomes than those with no evidence of congenital ZIKV infection aOR 6.7 (95% CI: 2.2–20.0) [11].

For those in our study with no evidence of ZIKV infection, or for whom ZIKV infection was not able to be determined, we are unable to infer whether this reflects: an absence of placental infection or in utero ZIKV infection; the waning of antibodies in early childhood; the inability of the developing fetal immune system to mount an immune response if infected early in the perinatal period; and/or an antibody response that is not easily detectable by the immunoassays used in this study. These findings highlight the need for further investigations into antibody kinetics in the perinatal and early childhood periods, and how these may correlate with the detection of abnormalities, in order to provide revised definitions of in utero ZIKV infection.

What remains unclear are the reasons for heterogeneity in rates of infection and abnormalities in observational studies to date. It also remains unclear whether immunological differences may partially explain the apparent differences in disease outcomes following ZIKV infection. For example, secondary infection with a heterologous DENV serotype is well established as a risk factor for severe DENV disease, which may be explained by cross-reactive antibodies that enhance DENV infection, known as antibody-dependent enhancement (ADE) [24,25]. For ZIKV, the role of prior flavivirus infection, particularly the role of the closely related DENV, which shares antigenic similarities with ZIKV, remains unclear. Studies have shown that the prior DENV infection may protect against ZIKV [26,27]. Other studies have found evidence for ADE in vitro [28], but little in vivo [29], or in epidemiological studies [30]. A reconciliation of the two hypotheses may lie in the timing of exposure to DENV and in the DENV serotype which may contribute to the differences observed in the strength of the relation between ZIKV infection and fetal abnormalities [31]. Our study involved participants living primarily in Guadeloupe and Martinique—French Territories in the Americas in which DENV is considered endemic, with co-circulation of more than one serotype [32] and where DENV seroprevalence exceeds 90% [32]. The extent to which flavivirus immunological differences may explain lower rates of infection and abnormalities in our study compared to other cohort studies warrants further investigation.

Underlying our findings is the assumption that all selected abnormalities were attributable to in utero ZIKV exposure. However, early childhood developmental trajectories are also influenced by genetic and environmental factors [33,34]. This is a critical consideration as a separate comparison of toddlers from the same study population who were exposed to ZIKV in utero with those who were not exposed recently found that while 15.4% of in utero exposed toddlers had possible neurodevelopmental delay at 24 months of age, there were minimal differences compared to ZIKV-unexposed toddlers [13]. Further, an analysis of infants born to women who had no evidence of prior ZIKV infection at the time of delivery found no statistically significant differences in the risk of neurological birth defects in live-born infants compared to infants born to women with ZIKV infection during pregnancy [35]. A population-based cohort study in Brazil found mortality risk to be higher among live-born children with congenital Zika syndrome as compared to those without and the risk was found to persist throughout early childhood [10]. These studies highlight the importance of an appropriate control group to avoid overattributing adverse outcomes to perinatal ZIKV infection.

Our findings are limited by our inability to include in the pediatric cohort study all infants born to women with symptomatic, RT-PCR-confirmed ZIKV infection during pregnancy and by the loss to follow-up from delivery to 24 months of age. However, the consultation of medical registries provided additional information as to the clinical status at 24 months of those not included in the pediatric cohort and those who were lost to follow-up. We are unable to determine the direction of possible selection bias among those who remained in the cohort study. That is infants with and without abnormalities declined to participate or were lost to follow-up. As such, it is unclear whether the absence of those infants may have resulted in an artificially over- or under-estimated risk of adverse pediatric outcomes.

## 5. Conclusions

Overall, our findings provide an additional contribution to understanding the risk of adverse pregnancy and early childhood outcomes following ZIKV infection during pregnancy. They provide evidence that will be helpful to clinical decisions regarding pregnancy management in the context of ZIKV infection. While many studies evaluating the impact of ZIKV infection during pregnancy have focused on adverse pregnancy outcomes, our study highlights the need for continued follow-up of infants born to women with ZIKV infection in pregnancy into childhood.

## Figures and Tables

**Table 1:** Characteristics of 546 women with RT-PCR-confirmed ZIKV infection during pregnancy and 527 liveborn infants.

1A. Maternal Characteristics	*n* = 546
Age at time of pregnancy (years)	
Mean ± SD	29.7 ± 6.2
Interquartile range	25.0–34.3
Occupation—*n* (%)	
Student	23 (4.2)
Self-employed/ business owner/ farmer	31 (5.7)
Executive/ highly skilled worker	55 (10.1)
Intermittent profession	56 (10.3)
Salaried employee	181 (33.2)
Not employed	188 (34.4)
Unknown or declined to respond	12 (2.2)
Educational attainment of mother—*n* (%)	
Primary	18 (3.3)
Secondary	38 (7.0)
Tertiary	60 (11.0)
Unknown or declined to respond	429 (78.7)
Educational attainment of father—*n* (%)	
Primary	11 (2.0)
Secondary	33 (6.0)
Tertiary	27 (4.9)
Unknown or declined to respond	475 (87.0)
Residence—*n* (%)	
Guadeloupe	245 (44.9)
Martinique	277 (50.7)
French Guiana	24 (4.4)
Previous pregnancies—*n* (%)	
0	131 (24.0)
1	150 (27.5)
2	128 (23.4)
3+	137 (25.1)
Previous adverse pregnancy outcomes—*n* (%)	
Congenital abnormalities	6 (1.1)
Stillbirth	10 (1.8)
Medical termination of pregnancy	10 (1.8)
Lifestyle practices during 2016–2017 pregnancy—*n* (%)	
Alcohol consumption	2 (0.4)
Drug use	6 (1.1)
Smoking	23 (4.2)
Use of mosquito repellents	445 (81.5)
Use of larvicides	337 (61.7)
**1B. Infant Characteristics**	***n* = 527**
Gestational age (weeks)	
Mean ± SD	38.7 ± 1.9
Delivery type—*n* (%)	
Cesarean	95 (18.0)
Sex—*n* (%)	
Male	266 (50.5)
Birth weight (g)	
Mean ± SD	3112 ± 530

**Table 2:** Selected adverse early childhood outcomes in 320 infants included in the ZIKA-DFA-BB cohort study across 24-month follow-up by ZIKV infection status.

	Consultation at 2 M *n* = 262 (%)			Consultation at 4 M *n* = 261 (%)			Consultation at 9 M *n* = 235 (%)			Consultation at 18 M *n* = 208 (%)			Consultation at 24 M *n* = 206 (%)			Consultation at Any Time across 24 M *n* = 310 (%)		
ZIKV Infection Status of Child	+ve *n* = 4	−ve *n* = 134	UD *n* = 124	+ve *n* = 4	−ve *n* = 138	UD *n* = 119	+ve *n* = 4	−ve *n* = 128	UD *n* = 103	+ve *n* = 4	−ve *n* = 130	UD *n* = 74	+ve *n* = 4	−ve *n* = 144	UD *n* = 58	+ve *n* = 5	−ve *n* = 162	UD *n* = 143
Hearing loss or deficiency *	0 (0)	4 (3.0)	3 (2.4)	0 (0)	0 (0)	0 (0)	0 (0)	0 (0)	1 (1.0)	1 (25.0)	0 (0)	0 (0)	0 (0)	1 (0.7)	1 (1.7)	1 (20.0)	5 (3.1)	5 (3.5)
Visual impairment **	1 (25.0)	8 (6.0)	3 (2.4)	0 (0)	1 (0.7)	2 (1.7)	1 (25.0)	0 (0)	1 (1.0)	1 (25.0)	0 (0)	1 (1.4)	0 (0)	2 (1.4)	1 (1.7)	2 (40.0)	11 (6.8)	8 (5.6)
Seizures, excluding febrile seizures	0 (0)	0 (0)	0 (0)	0 (0)	0 (0)	0 (0)	0 (0)	0 (0)	0 (0)	0 (0)	1 (0.8)	1 (1.4)	0 (0)	1 (0.7)	2 (3.4)	0 (0)	1 (0.6)	2 (1.4)
Body tone, musculoskeletal or motor abnormalities	0 (0)	3 (2.2)	1 (0.8)	1 (25.0)	5 (3.6)	3 (2.5)	1 (25.0)	2 (1.6)	1 (1.0)	1 (25.0)	2 (1.5)	1 (1.4)	1 (25.0)	1 (0.7)	3 (5.2)	2 (40.0)	9 (5.6)	6 (4.2)
Swallowing abnormalities	0 (0)	12 (9.0)	6 (4.8)	0 (0)	0 (0)	1 (0.8)	-	-	-	-	-	-	-	-	-	0 (0)	12 (7.4)	7 (4.9)
Post-natal microcephaly	0 (0)	0 (0)	0 (0)	1 (25.0)	0 (0)	0 (0)	0 (0)	1 (0.8)	0 (0)	1 (25.0)	1 (0.8)	0 (0)	1 (25.0)	1 (0.7)	0 (0)	1 (20.0)	1 (0.6)	0 (0)
Any selected abnormality ***	1 (25.0)	23 (17.2)	12 (9.7)	1 (25.0)	6 (4.3)	5 (4.2)	1 (25.0)	3 (2.3)	2 (1.9)	1 (25.0)	4 (3.1)	3 (4.0)	1 (25.0)	6 (4.2)	5 (8.6)	3 (60.0)	36 (22.2)	24 (16.7)
Imaging abnormality ^+^	2 (50.0)	11 (8.2)	10 (8.1)	0 (0)	1 (0.7)	0 (0)	0 (0)	0 (0)	0 (0)	-	-	-	-	-	-	2 (40.0)	12 (7.4)	10 (7.0)
Any selected abnormality, including imaging	3 (75.0)	34 (25.4)	21 (16.9)	1 (25.0)	7 (5.1)	5 (4.2)	1 (25.0)	3 (2.3)	2 (1.9)	1 (25.0)	4 (3.1)	3 (4.1)	1 (25.0)	6 (4.2)	5 (8.6)	3 (60.0)	43 (26.5)	33 (23.1)

ZIKV infection status of child: +ve: in utero ZIKV infection confirmed; −ve: in utero ZIKV infection excluded; UD: in utero ZIKV infection confirmed unknown/undetermined. * Includes results of screening evaluation, and additional auditory examination. ** Includes results of screening evaluation, and additional ophthalmologic examination. *** Any selected abnormality: Hearing loss or deficiency; Visual impairment; Seizures, excluding febrile seizures; Body tone, musculoskeletal or motor abnormalities; Swallowing abnormalities; Post-natal microcephaly. + Imaging abnormality: abnormal transfontanelle cerebral ultrasound, and/or MRI.

**Table 3:** Severity of adverse pregnancy and early childhood outcomes * by trimester of maternal ZIKV infection.

3A	Trimester of Maternal ZIKV Infection			All *n* = 555 (%)
Severity of Adverse Pregnancy and Early Childhood Outcomes	1 *n* = 158 (%)	2 *n* = 257 (%)	3 *n* = 140 (%)	
Severe sequelae or fatality	24 (15.2)	11 (4.3)	2 (1.4)	37 (6.7)
Major abnormalities	7 (4.4)	9 (3.5)	8 (5.7)	24 (4.3)
Mild abnormalities	23 (14.6)	38 (14.8)	23 (16.4)	84 (15.1)
No abnormality reported	90 (57.0)	171 (66.5)	95 (64.1)	356 (64.1)
Unknown	14 (8.9)	28 (10.9)	12 (8.6)	54 (9.7)
**3B**	**Trimester of Maternal ZIKV Infection**			**All** ***n* = 555 (%)**
Severity of Adverse Pregnancy and Early Childhood Outcomes Related to Maternal ZIKV Infection **	**1** ***n* = 158 (%)**	**2** ***n* = 257 (%)**	**3** ***n* = 140 (%)**	
Severe sequelae or fatality	11 (7.0)	7 (2.7)	2 (1.4)	20 (3.6)
Major abnormalities	3 (1.9)	8 (3.1)	4 (2.9)	15 (2.7)
Mild abnormalities	15 (9.5)	19 (7.4)	18 (12.9)	52 (9.4)
No abnormality reported	103 (65.2)	195 (75.9)	104 (74.3)	402 (72.4)
Unknown	26 (16.5)	28 (10.9)	12 (8.6)	66 (11.9)

* Categorization of severity of abnormalities available in Supplementary Table S2. ** Selected adverse pregnancy and early childhood outcomes as per 3A, with the following reclassification: Miscarriages = Unknown; stillbirths without abnormalities = No abnormality reported. In addition, non pathological findings for vision (retinal hemorrhages at birth) were considered ‘no vision abnormality’ and non pathological imaging findings were considered ‘no imaging abnormality’, with categorization of the severity of abnormalities for these infants following the categorization of abnormalities described in Supplementary Table S2.

## Data Availability

The deidentified participant data presented in this study are available upon reasonable request to the corresponding author. The data are not publicly available.

## Supplementary Materials

The following supporting information can be downloaded at: https://www.mdpi.com/article/10.3390/v14122755/s1, Table S1. A. Maternal and B. Neonatal characteristics of 315 women with symptomatic, RT-PCR confirmed ZIKV infection during pregnancy who included their infant in pediatric cohort study and 203 women with symptomatic, RT-PCR confirmed ZIKV infection during pregnancy who declined participation of their infant in the pediatric cohort study. Table S2. Categorization of severity of abnormalities.
