# Supplementary material for: Consequences of In Utero Zika Virus Exposure and Adverse Pregnancy and Early Childhood Outcomes: A Prospective Cohort Study
Source: Viruses. 2022 Dec 10;14(12):2755. doi: 10.3390/v14122755 (PMC9788325; doi:10.3390/v14122755)
Supplement: Supplementary file 1 [file viruses-14-02755-s001.zip › viruses-2080602-supplementary.pdf]

**Consequences of in utero Zika virus exposure and adverse pregnancy and early childhood outcomes: a prospective cohort study**

**Supplementary Material**

**Supplementary Table S1.** A. Maternal and B. Neonatal characteristics of 315 women with symptomatic, RT-PCR confirmed ZIKV infection during pregnancy who included their infant in pediatric cohort study and 203 women with symptomatic, RT-PCR confirmed ZIKV infection during pregnancy who declined participation of their infant in the pediatric cohort study

| A. Maternal characteristics | Mothers of infants included in pediatric cohort study N=315 (%) | Mothers of infants not included in pediatric cohort study N=203 (%) | p-value |
|---|---|---|---|
| Age at time of pregnancy (years) | | | |
| Mean ±SD | 30.0 ± 6.4 | 29.1 ± 5.9 | 0.10 |
| Interquartile range | 25.4 – 34.7 | 24.8 – 33.4 | |
| Occupation – n (%) | | | 0.20 |
| Student | 12 (3.8) | 9 (4.8) | |
| Self-employed/ business owner/ farmer | 15 (4.8) | 16 (6.9) | |
| Executive/ highly skilled worker | 31 (9.8) | 19 (10.4) | |
| Intermittent profession | 33 (10.5) | 18 (10.0) | |
| Salaried employee | 120 (38.1) | 57 (26.4) | |
| Not employed | 97 (30.8) | 79 (39.3) | |
| Unknown or declined to respond | 7 (2.2) | 5 (2.2) | |
| Educational attainment of mother – n (%) | | | |
| Primary | 18 (5.7) | - | |
| Secondary | 38 (12.1) | - | |
| Tertiary | 60 (19.0) | - | |
| Unknown or declined to respond | 199 (63.2) | - | |
| Educational attainment of father – n (%) | | | |
| Primary | 11 (3.5) | - | |
| Secondary | 33 (10.5) | - | |
| Tertiary | 27 (8.6) | - | |
| Unknown or declined to respond | 244 (77.5) | - | |
| Residence – n (%) | | | *<0.001* |
| Guadeloupe | 139 (44.1) | 98 (45.9) | |
| Martinique | 175 (55.6) | 85 (45.0) | |
| French Guiana | 3 (1.0) | 20 (9.1) | |
| Parity – n (%)* | | | 0.97 |
| 0 | 75 (23.8) | 52 (25.6) | |
| 1 | 86 (27.3) | 53 (26.1) | |
| 2 | 73 (23.2) | 46 (22.7) | |
| 3+ | 81 (25.7) | 52 (25.6) | |
| Previous adverse pregnancy outcomes – n (%) | | | |
| Congenital abnormalities | 4 (1.3) | 2 (0.9) | 1 |
| Stillbirth | 7 (2.2) | 1 (0.9) | 0.23 |
| Medical termination of pregnancy | 3 (1.0) | 6 (3.0) | 0.17 |
| Lifestyle practices during 2016-2017 pregnancy – n (%) | | | |
| Alcohol consumption | 0 (0) | 1 (0.9) | 0.82 |
| Drug use | 3 (1.0) | 3 (1.3) | 0.89 |
| Smoking | 11 (3.5) | 12 (5.3) | 0.28 |
| Use of mosquito repellents | 259 (82.7) | 165 (81.6) | 0.94 |
| Use of larvicides | 193 (62.3) | 123 (65.2) | 0.87 |
| **B. Neonatal characteristics** | **Included in pediatric cohort study N=320 (%)** | **Not included in pediatric cohort study N=207 (%)** | **p-value** |
| Gestational age (weeks) | | | |
| Mean ±SD | 39.0 ± 1.4 | 38.4 ± 2.4 | *0.002* |

| | | | | |
|---|---|---|---|---|
| Delivery type – n (%)* | | | | |
| | Cesarean | 60 (18.8) | 35 (17.8) | 0.81 |
| Sex – n (%) | | | | |
| | Male | 163 (50.9) | 103 (48.9) | 0.95 |
| Birth weight (g) | | | | |
| | Mean | 3159 ± 466 | 3039 ± 612 | *0.02* |

**Supplementary Table S2.** Categorization of severity of abnormalities

| |
|---|
| **Severe sequelae or fatal outcome** |
| Not liveborn or post-natal death |
| Multiple and persistent sequelae, abnormal imaging |
| Multiple and persistent sequelae, normal imaging |
| Isolated and persistent non-febrile seizures |
| Spina bifida at birth (alive at 24M, no abnormalities reported) |
| Club foot at birth (alive at 24M, no abnormalities reported) |
| Club foot at birth (no information after birth) |
| **Major abnormalities** |
| One selected abnormality and abnormal imaging |
| Two isolated selected abnormalities, normal imaging |
| Two isolated selected abnormalities, no imaging information |
| Microcephaly at birth, lost to follow up (no information after birth) |
| Isolated and persistent post-natal onset microcephaly |
| Isolated, single occurrence non-febrile seizures (at 24M) |
| **Mild abnormality** |
| Isolated abnormal imaging (+ transient swallowing abnormality at 2M) |
| Isolated eye abnormality (+ transient swallowing abnormality at 2M) |
| Single isolated abnormality |
| Abnormal imaging (no other abnormality) |
| Isolated microcephaly at birth (+ transient swallowing abnormality at 2M) |
| **No abnormality reported** |
| Transient swallowing abnormality at 2M |
| Non-pathological imaging finding |
| No abnormalities reported |
| **Unknown** |
| Unknown (no abnormality information available) |
